# Same Habitat, Different Responses: Population Dynamics of Two Sympatric Invader *Corbicula* Species

**DOI:** 10.3390/biology15131026

**Published:** 2026-06-27

**Authors:** Gustavo Darrigran, Cristina Damborenea, Pablo Penchaszadeh, Darío Colautti, Miriam Maroñas, Diego E. Gutiérrez Gregoric

**Affiliations:** 1 División Zoología Invertebrados, Museo de La Plata, FCNyM-UNLP and CONICET, Paseo del Bosque, La Plata 1900, Argentina; diegoeguty@gmail.com; 2 Museo Argentino de Ciencias Naturales “Bernardino Rivadavia”, Av. Ángel Gallardo 470, Ciudad Autónoma de Buenos Aires C1405DJR, Argentina; pablopench@gmail.com; 3 Instituto de Limnología “Dr. Raúl A. Ringuelet” (ILPLA) (CONICET-UNLP), Boulevard 120 y 62, La Plata 1900, Argentina; colautti@ilpla.edu.ar (D.C.);

**Keywords:** biological invasions, population density, size structure, reproductive biology, South America, Pampean stream

## Abstract

Invasive species require long-term characterization of their shifting population dynamics to understand their environmental impacts and socio-economic effects. The dynamics of freshwater bivalves depend on their biology, which is influenced by the vulnerability of the ecosystems. This study evaluates the coexistence of *Corbicula fluminea* and *C. largillierti* in a stream in the Argentine Pampa, integrating density, size, and reproduction. In this stream, characterized by a temperate climate and hydrological fluctuations, live specimens of both species were sampled monthly using a 0.07 m^2^ cylinder. The anteroposterior length of each specimen was measured, and size distributions were analyzed by decomposing normal modes using the least-squares method. Gonadal cycles were compared with data from the previous literature. Since 2004, *C. fluminea* has dominated the system with significantly higher densities. Both species exhibited contrasting reproductive cycles. Hydrological instability in the stream limited both species. *C. fluminea* dominated due to its greater resilience and longevity under stress. In contrast, *C. largillierti* prioritized initial rapid growth, making it more sensitive to fluctuations. These results highlight that environmental instability conditions invasive success and interspecific competition in freshwater systems.

## Simple Summary

Effective management of bioinvasions not only requires documenting the presence of invasive species, but also understanding the broad range of their population dynamics over time (such as delayed reproduction, rapid population growth, and fluctuations in density). Several species of the genus *Corbicula* are among the most widespread invasive bivalves globally and have rapidly expanded throughout South America. This study aimed to evaluate the coexistence patterns between *Corbicula fluminea* and *C. largillierti*, considering key population dynamic parameters such as density, size structure, and reproductive biology in an unstable environment (affected by droughts and floods) within a Pampean stream in Argentina. Fluctuations in the stream’s water level and flow rate affected these species differently. *C. fluminea* dominated due to its resilience and longevity, while *C. largillierti* was more sensitive despite its initial rapid growth. We conclude that the environment thus shapes invasion success and interspecific competition.

## 1. Introduction

Understanding biological invasions not only requires documenting the presence of invasive species, but also characterizing their long-term population dynamics, as these ultimately determine ecological impacts and inform management strategies [1]. As invasions progress, changes in abundance and population traits can lead to shifts in both ecological impacts and economic effects [2]. Invasive populations are known to exhibit a wide range of dynamics, including delayed reproduction, rapid population growth, sudden collapses (even leading to local extinction), and long-term increases or declines in density; however, these dynamics remain poorly understood from both empirical and theoretical perspectives [3]. In bivalves, these complexities are further shaped by life-history strategies in which population dynamics, growth, and reproduction are tightly interconnected and strongly influenced by environmental variability [4]. Temporal fluctuations in density are often driven by recruitment pulses and mortality events, which in turn structure size distributions and cohort progression. At the same time, growth rates and size structure can modulate reproductive output, as reproduction is frequently size-dependent and energetically constrained [5,6]. In freshwater environments, these processes are regulated by external drivers such as hydrological disturbances, including floods and droughts, which affect resource availability, habitat stability, and physiological performance [7]. Consequently, the population trajectories and impacts of invasive freshwater bivalves are expected to arise from the interaction between intrinsic biological traits and environmentally driven constraints [8].

The genus *Corbicula* (Bivalvia: Cyrenidae) comprises both estuarine and freshwater species and represents the most species-rich genus within the family Cyrenidae [9]. Several species within this genus rank among the most widespread invasive bivalves worldwide [10] and have rapidly expanded across South America [11] since their first detection in the early 1980s [12]. *Corbicula* spp. frequently form dense populations and can induce substantial ecosystem changes, both through alterations of trophic webs and through their role as ecosystem engineers [13]. These impacts are evident, for example, in benthic macroinvertebrate communities, where they modify taxonomic composition and affect dominant taxa [14].

In the Neotropical region, at least four non-native *Corbicula* species have been reported to occur sympatrically: *C. largillierti* (Philippi, 1844), *C. fluminalis* (Müller, 1774), *C. fluminea* (Müller, 1774), and *Corbicula* sp. [15]. Among these, *C. fluminea* is generally considered to have higher competitive ability, often displacing congeneric species such as *C. largillierti* [16]. As a result, *C. fluminea* frequently becomes dominant in invaded habitats, acting as a strong ecosystem engineer and causing both physical and biological alterations [13]. However, such displacement patterns have been described for other invasive bivalves as dreissenids [17].

Reshaid et al. [18] compared density values of *C. fluminea* and *C. largillierti* along the Río de la Plata River shoreline during the 1980s, near the sites of the first record of the genus in South America, with those recorded approximately 25 years later (2015–2016) at the same locations. Their results showed a marked retreat of *Corbicula* spp. from this system. While in the 1980s *C. fluminea* and *C. largillierti* exhibited occurrence frequencies of 75% and 37%, respectively, *C. fluminea* showed a substantial decline in frequency in the later period, and no live individuals of *C. largillierti* were recorded. These differences were accompanied by a significant reduction in density, with *C. fluminea* decreasing by approximately 90% and *C. largillierti* effectively disappearing from the area.

Size structure can indicate whether an invasive species is expanding or declining, as it reflects the population state within the ecosystem [19]. For instance, a size distribution including both juveniles and adults suggests a stable population. In the Río de la Plata River (Argentina), early studies, in the late 1980s, showed that both *Corbicula largillierti* (between 1 and 31 mm total length) and *Corbicula fluminea* (between 1 and 36 mm) had size distributions dominated by small individuals (~2 mm), indicating expanding populations [20]. However, 25 years later, *C. largillierti* had disappeared, while *C. fluminea* persisted at lower densities and smaller average sizes (<20 mm) [18]. Although a high proportion of small individuals in *C. fluminea* could suggest ongoing recruitment and expansion, this interpretation is inconsistent with the invasion history. *Corbicula* species were introduced to the Río de la Plata in the late 1960s–early 1970s [12], indicating a long-established invasion.

Differences in habitat use and competitive ability have been proposed to explain these patterns. *Corbicula fluminea* is considered competitively superior and is dominant in large rivers and estuarine environments, whereas *C. largillierti* has been displaced toward tributary streams, where it may coexist with *C. fluminea*, albeit at lower densities [16]. This preference for lotic environments has also been reported in other basins, such as the Suquía River [21]. Currently, *C. fluminea* is widely distributed in the Río de la Plata River and major rivers of eastern and southern Argentina, where it shows greater adaptive capacity, while *C. largillierti* predominates in smaller rivers, streams, and lakes in central and northwestern regions [22]. Environmental factors further contribute to shaping these distribution patterns. The density of invasive bivalves such as *C. fluminea* is influenced by substrate composition, slope, and water characteristics [23]. In particular, its presence has been positively associated with sandy sediments and is unlikely in environments where sand content falls below 66% [24].

The reproductive strategy used is another key factor that influences the success of invasive species in novel environments [9,25]. The genus *Corbicula* exhibits a remarkable diversity of reproductive modes, including both sexual reproduction and androgenesis. The sexual structure of populations varies geographically, ranging from dioecious systems in native ranges to predominantly hermaphroditic populations in invaded areas, often with variable sex ratios [26,27,28]. While some species, such as *C. sandai* and *C. japonica*, reproduce via external fertilization through the release of free gametes, a condition considered ancestral among freshwater bivalves [29], many invasive lineages exhibit derived reproductive traits. In particular, *Corbicula* spp. are typically protogynous [29]. For example, *C. largillierti* in South America is described as a simultaneous hermaphrodite [30]. Self-fertilization, well documented in *C. fluminea* [31], has also been proposed for *C. largillierti* in the region [32]. Foundational studies addressing reproductive strategies of *Corbicula* in South America include Ituarte [30,32], Mansur et al. [33], Cao et al. [34], Dei Tos et al. [35], and Damborenea et al. [36].

In this context, the present study aims to evaluate the coexistence of *Corbicula fluminea* and *C. largillierti* under sympatric conditions by integrating population density, size structure, and reproductive traits in a Pampean stream in Argentina. From January 2003 to April 2005, both species were monitored concurrently, allowing for a direct comparison of their reproductive cycles and population dynamics within the same environmental setting [34,36]. This coexistence provides an opportunity to evaluate whether closely related species exhibit similar or divergent ecological responses under shared environmental conditions. We hypothesize that the dominance of *C. fluminea* reflects its ability to preserve coordinated growth, reproduction, and population dynamics under environmental variability, in contrast to *C. largillierti*, where this coordination is lost under stressful conditions.

## 2. Materials and Methods

### 2.1. Study Area

**Figure 1:**
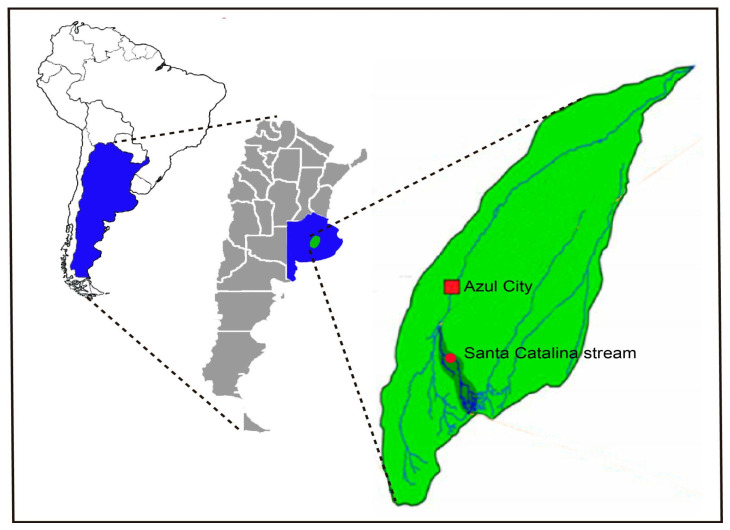
Geographical location of the Santa Catalina stream. Light green inset represents the Azul basin, and dark green the Santa Catalina stream basin. Red dot indicates the sampling site (36°53′04.5″ S; 59°55′25.22″ W). The color blue indicates Argentina (on the map of South America) and the Buenos Aires Province (on the map of Argentina in the center of the figure).

The study was conducted in the Santa Catalina stream, Buenos Aires Province, Argentina (Figure 1). The stream basin covers an area of 138 km^2^, and the sampling site was located in the lower basin at 151 m above sea level, within the “Pampa Deprimida” region. This area is characterized by flat topography, low relief, and a poorly developed drainage system [37]. Climatic conditions include maximum temperatures between December and March (monthly mean: 20 °C) and minimum temperatures between June and August (monthly mean: 8 °C). Annual precipitation averages approximately 840 mm, with higher rainfall during spring–summer (~100 mm/month) and lower values in autumn–winter (~50 mm/month). The region is subject to recurrent hydrological fluctuations, including both droughts and floods [37].

Physical and chemical parameters (i.e., water temperature, dissolved oxygen, oxygen saturation, conductivity, and total dissolved solids) were measured in the field using a Lutron WA-2017SD multi-parameter water quality meter. Daily rainfall, streamflow, and water level data were provided by the Instituto de Hidrología de Llanuras “Dr. Eduardo Jorge Usunoff” (IHLLA) in Azul, Argentina. Limnological variables recorded during the study period are reported in Cao et al. [34] and Damborenea et al. [36] (Table 1). Mean flow showed differences between the first (2003) and second (2004–2005) sampling periods.

### 2.2. Field Sampling

All samples were collected at Santa Catalina stream and its intersection with National Route 3 (36°53′04.5″ S; 59°55′25.22″ W) (Figure 1). Sampling was always carried out in the presence of water in the environment. Twenty-seven samples were collected between January 2003 and April 2005 [34,36]. Sampling was performed using a cylindrical corer (0.07 m^2^), which was manually inserted into the sediment to a depth of approximately 0.20 m (until reaching the compact sediment layer). Three replicate samples were taken on each date. Samples were sieved in situ using a 1 mm mesh, and live specimens of *Corbicula largillierti* and *C. fluminea* were transported to the laboratory for analysis. The specimens were identified according to valve morphology [30,36]. As *Corbicula* spp. are invasive in the Neotropical region, no specific permits were required for their collection.

### 2.3. Genetic Analysis

To confirm the morphological identification of both *Corbicula* species, genomic DNA of three specimens identified as *C. fluminea* and of two identified as *C. largillierti* was extracted from a tissue sample taken from the foot of specimens collected in Santa Catalina stream, using the commercial Wizard Promega kit, Promega Corporation, Madison, WI, USA (www.promega.com, accessed 22 June 2026). A partial fragment of the COI gene was amplified by polymerase chain reaction (PCR) using universal primers [38]. Amplification was performed in a final volume of 25mL following the protocol described by [39]. The PCR products were purified, and sequencing of both DNA strands was carried out by Macrogen (Seoul, Republic of Korea). Raw sequences were edited in BioEdit version 7.0.5.3, University of North Texas, Denton, TX, USA [40] and aligned with homologous COI sequences from GenBank using Clustal X version 2.0.12 [41]. The resulting sequences were compared with reference sequences from GenBank, https://www.ncbi.nlm.nih.gov/genbank/ (accessed on 22 June 2026) and with data from previous studies conducted in Argentina and South America [42,43] using the BLASTN algorithm [44]. Corrected genetic distances (using the Kimura 2-parameter model) were calculated with MEGA X [45].

### 2.4. Size Structure and Density

A total of 5969 live individuals of *Corbicula fluminea* and 4913 of *C. largillierti* were analyzed. The maximum anteroposterior length (APL) of each specimen was measured using a digital caliper Mitutoyo Corporation, Jundiaí, Brazil, accurate to 0.1 mm. Mean density (individuals/m^2^) and standard deviation were calculated for each sampling date. Population size structure was assessed using 1 mm APL size classes between January 2003 and September 2004. Specimens smaller than 3 mm were excluded from the analysis because species cannot be reliably distinguished morphologically at smaller sizes. To test for differences in density between the populations of both *Corbicula* species, a Wilcoxon signed-rank test was applied [46].

The polymodal APL frequency distributions for each date and species using 1mm class intervals, were decomposed into their normal components (assuming a normal length distribution for each cohort) to obtain the mean APL, standard deviation, and density of each detected cohort [47]. The procedure for the decomposition consisted of finding, using the least squares method, the sum of normals that best fit the observed polymodal distribution (R^2^ > 0.95), using a spreadsheet programmed in Excel and its Solver add-in.

### 2.5. Gonadal and Life Cycle Comparison with Environmental Variables

Previous studies on the reproductive biology of *Corbicula fluminea* [34] and *C. largillierti* [36] were compared. This was possible because both investigations were carried out using the same methodology (histological analysis) and correspond to the same sampling site and dates of the present research.

Spearman’s correlations were performed to determine the relationship between the percentage of presence of different gonadal stages and the presence of individuals with incubating larvae and physical and chemical environmental parameters. Canonical correspondence analysis (CCA) was performed to compare qualitative gonadal stages, oocyte size, and environmental parameters using PAST software version 4.17c [48]. Environmental variables included in the analysis were conductivity (µS/cm), mean water level (m), oxygen concentration (mg/L), mean flow rate (m^3^/s), and water temperature (°C).

## 3. Results

### 3.1. Molecular Validation of Corbicula Species Identification

COI gene sequences were obtained from five specimens, confirming the coexistence of both species within the same environment. Three sequences showed a 100% match with haplotype FW5, corresponding to the form AR of Corbicula fluminea. The remaining two sequences showed a 100% match with haplotype FW17, corresponding to the form CS of *Corbicula largillierti* (Table 2 and Table 3). The sequences generated in this study were deposited in GenBank.

### 3.2. Density and Size

**Figure 2:**
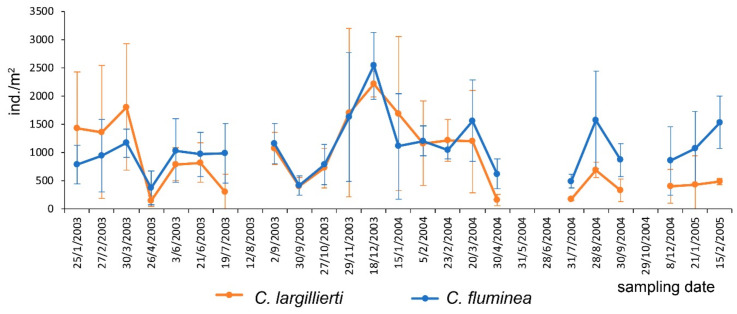
Mean density (ind./m^2^) of *C. fluminea* and *C. largillierti* in Santa Catalina stream during the sampled period. Lines denotes ± 1 standard deviation.

A total of 5969 specimens of *Corbicula fluminea* and 4913 of *Corbicula largillierti* were collected from the Santa Catalina stream. Although the density of both species exhibited marked temporal variability (Figure 2), no statistically significant differences were detected between them. The Wilcoxon signed-rank test indicated a small, non-significant difference between *C. largillierti* (Mdn = 1199, *n* = 47) and *C. fluminea* (Mdn = 1038, *n* = 47) (Z = −1, *p* = 0.304, r = −0.2). At the beginning of the study period (January 2003), the density of *C. largillierti* reached 1777 ind./m, whereas *C. fluminea* showed a lower density of 788 ind./m^2^. By the end of the sampling period (March 2005), this pattern had reversed, with *C. fluminea* attaining a density of 1100 ind./m^2^ and *C. largillierti* declining to 228 ind./m^2^.

**Figure 3:**
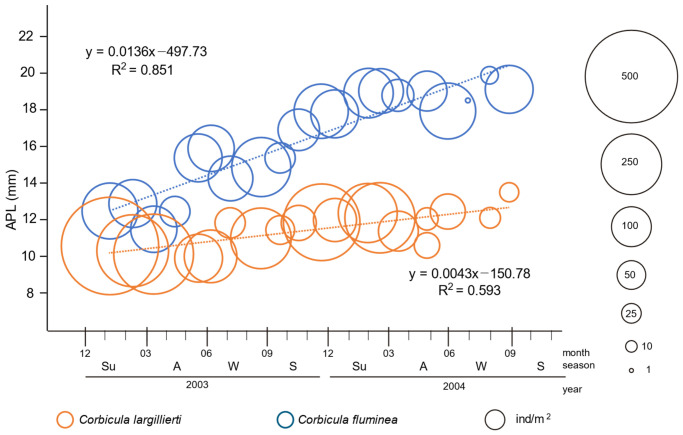
Temporal tracking of total length (mm) and density of the dominant size classes for *C. fluminea* (blue) and *C. largillierti* (orange) over the period January 2003–September 2004. Circle diameter represents the density of individuals at each sampling date. Linear trend lines are shown, along with their corresponding equations and coefficients of determination (R^2^).

The decomposition of size-frequency distributions for each sampling date and species generally revealed the presence of a single dominant size group. Given this pattern, we tracked changes in the size and density of the components of these dominant size classes (Figure 3).

Thus, the decomposition of size distributions at each date was characterized by a dominant group of individuals in terms of density, whose sizes showed a progressive increase and, in general, a decrease in numerical representation over the course of the sampling period.

Differences in growth between the two species were evident throughout the sampling dates. *C. largillierti* showed a moderate fit (R^2^ = 0.59), indicating greater size variability compared to *C. fluminea*. In contrast, the growth of *C. fluminea* was more consistent over time, with an R^2^ of 0.85. Moreover, *C. largillierti* not only exhibited generally lower densities, but also a markedly lower growth rate than *C. fluminea*, with a slope of 0.0043, indicating a growth rate nearly three times lower than that of *C. fluminea*, which showed an average growth rate of 0.0136 over the same period.

Additionally, *C. largillierti* reached average sizes of approximately 13–14 mm by the end of the study period, with a maximum size in the environment of about 19.20 mm. In contrast, *C. fluminea* exhibited modal sizes around 20 mm in the final sampling period (Figure 3), reaching maximum sizes in the environment of approximately 27–28 mm (Table 4) (Figures S1 and S2).

In Figure 3, regarding the relative abundance of cohorts, both species showed substantial recruitment toward the end of 2003 (large bubbles at small sizes). As time progressed, cohorts of *C. fluminea* displayed a clearer progression, whereas the modes of *C. largillierti* remained more clustered.

### 3.3. Reproduction

**Figure 4:**
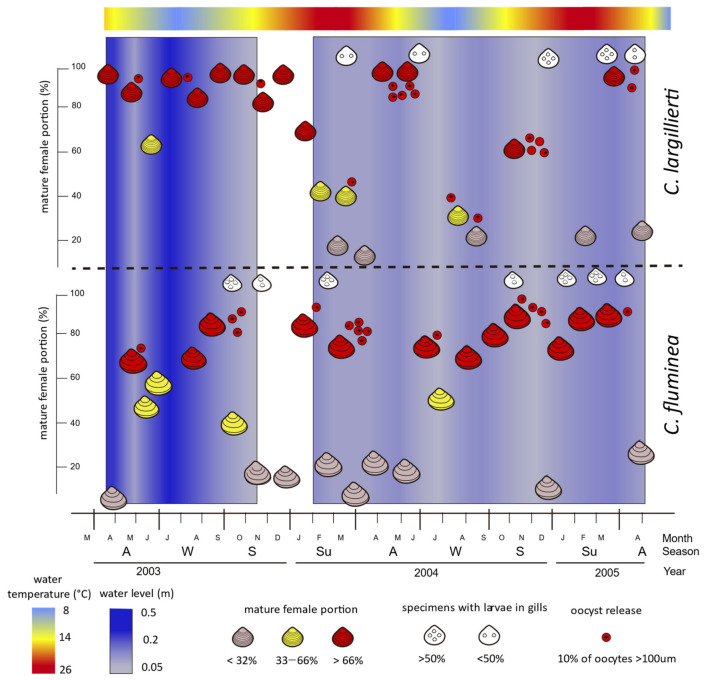
Reproductive dynamics of *Corbicula fluminea* and *Corbicula largillierti* between 2003 and 2005 [34,36]. The proportion of mature female follicles (%) over time is shown, with sampling months grouped by seasons (Su: summer; A: autumn; W: winter; S: spring), together with the occurrence of oocyte release and the presence of larvae in the gills. Symbol colors indicate proportion of maturity (beige: <32%; yellow: 33–66%; red: >66%). Small red circles represent oocyte release events, whereas white symbols indicate the proportion of individuals with incubated larvae in the gills (>50% or <50%). The color gradient in the upper bar represents water temperature (°C), and the background shading (from blue to gray) indicates water level in the Santa Catalina stream. The year 2003 corresponds to a period of environmental stress, characterized by increased water level and water flow (intense blue), whereas 2004–2005 represent more stable water level (light blue). Differences in clam size reflect the maximum size reached by each species.

Populations of *Corbicula fluminea* and *C. largillierti* exhibited contrasting patterns in their reproductive cycles (Table 5, Figure 4). In *C. fluminea*, the pattern was relatively constant over time, with high proportions of mature female follicles (generally close to 80%) throughout most of the study period. Peaks in maturity were associated with marked oocyte release events, mainly during spring and summer, although lower-intensity releases were also recorded during autumn and winter.

In contrast, *Corbicula largillierti* showed more pronounced variation among periods. During the phase of high water level (2003), high proportions of mature follicles were observed (often close to 100%), but with limited oocyte release. Conversely, during the period of hydrological stability (2004–2005), the proportion of mature follicles was more variable, and the main oocyte release events were recorded. This suggests a decoupling between maturation and release under stressful conditions, followed by a reactivation of reproductive activity under more favorable conditions.

Additionally, differences in larval incubation were observed. In *C. fluminea*, the presence of larvae in the gills was continuous across both periods, whereas in *C. largillierti* it was only recorded during the phase of environmental stability.

### 3.4. Relationship Between Physical and Chemical Parameters with Gonadal Stages

**Figure 5:**
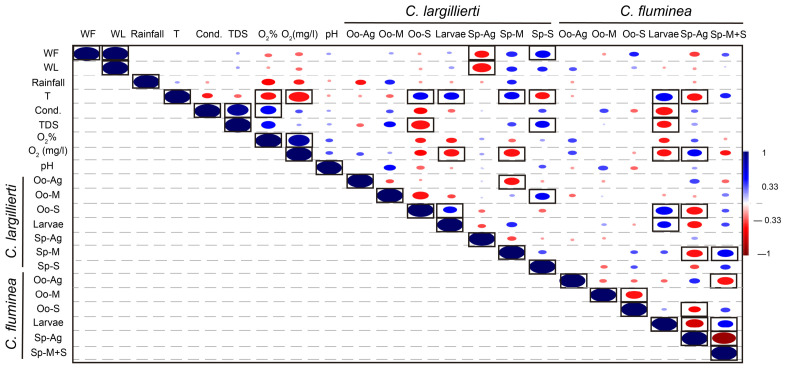
Correlation between environmental variables and gonadal stages of *C. largillierti* and *C. fluminea*. Environmental variables: COND, conductivity (uS/cm); O_2_, oxygen concentration (mg/L); O_2_ %, percentage of oxygen; pH; Rainfall (mm); T, temperature (°C); TDS, total dissolved solids (mg/L); WF, water flow (m^3^/s); WL, water level (m); gonadal stages: Larvae, proportion of individuals brooding larvae in the gills; Oo-AG, oogenic follicles in active gametogenesis; Oo-M, mature oogenic follicles; Oo-S, spawned oogenic follicles; Sp-AG, spermatogenic follicles with active gametogenesis; Sp-M, mature spermatogenic follicles; Sp-S, spawned spermatogenic follicles. In blue, positively correlated; in red, negatively correlated; circles in boxes, significantly correlated.Canonical correspondence analysis showed relations among physical and chemical parameters with the reproductive characteristics. For *C. largillierti*, the first two axes explained 96.23% of the variability, with axis 2 being clearly significant. High temperatures are related to the evacuation of oogenetic follicles and the maturation of spermatogenetic follicles. On the other hand, high streamflow and stream level values are related to the evacuation of spermatogenetic follicles and, to a lesser extent, to their maturation. Conductivity is related to the maturity of oogenetic follicles, while active gametogenesis shows no marked environmental association (Figure 6).

Gonadal stages were correlated with physical and chemical parameters. In *C. largillierti*, the proportion of spent female follicles increased significantly with rising temperature (*S* = 0.467, *p* < 0.033) and decreasing total dissolved solids (*S* = −0.667, *p* = 0.002). Similarly, maturing spermatogenetic follicles correlated positively with temperature (S = 0.477, *p* < 0.029) and negatively with dissolved oxygen (*S* = −0.581, *p* < 0.007). While the proportion of spawned spermatogenetic follicles correlated with increasing water flow (*S* = 0.488, *p* < 0.016) and decreasing temperature (*S* = −0.456, *p* < 0.038), male follicles in active gametogenesis showed negative correlations with both water flow and water level (*S =* −0.462, *p* < 0.023 and *S* = −0.643, *p* < 0.001, respectively). Conversely, in *C. fluminea*, spermatogenetic follicles in active gametogenesis only correlated significantly with decreasing temperature (*S* = −0.478, *p* < 0.028) and increasing dissolved oxygen (*S* = 0.481, *p* < 0.032). For both species, the proportion of individuals brooding larvae in the gills was positively related to temperature (*C. largillierti*: *S* = 0.521, *p* < 0.015; *C. fluminea*: *S* = 0.595, *p* < 0.004) and inversely correlated with dissolved oxygen (*C. largillierti*: *S* = −0.459, *p* < 0.042; *C. fluminea*: *S* = −0.474, *p* < 0.035). Additionally, larval presence in *C. fluminea* was negatively correlated with conductivity (*S* = −0.609, *p* < 0.007) and total dissolved solids (*S* = −0.524, *p* < 0.026) (Figure 5).

**Figure 7:**
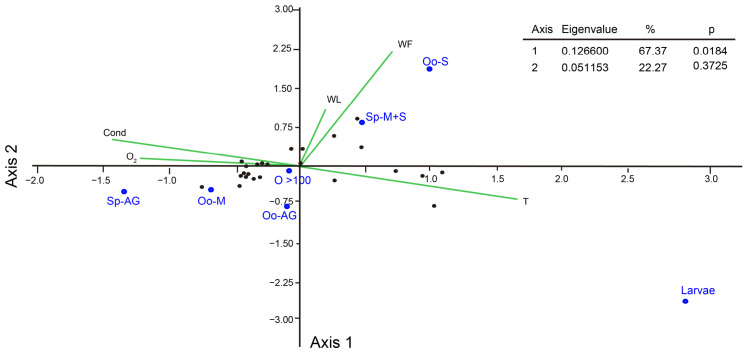
Canonical correspondence analysis triplot (scaling type 2). Black dots, samples. Blue dots, reproductive characteristics of *Corbicula fluminea*: O > 100, oocytes bigger than 100 um; Larvae, proportion of individuals brooding larvae in the gills; Oo-AG, oogenic follicles in active gametogenesis; Oo-M, mature oogenic follicles; Oo-S, spawned oogenic follicles; Sp-AG, spermatogenic follicles with active gametogenesis; Sp-M+S, mature and spawned spermatogenic follicles. Green lines, environmental variables: Cond, conductivity (uS/cm); WL, mean water level (m); O_2_, oxygen concentration (mg/L); WF, water flow (m^3^/s); T, temperature (°C).

For *C. fluminea*, the first two components explained 89.64% of the variability, with axis 1 being significant. Waterflow, stream water level, and temperature are related to maturing and spawning spermatogenic follicles, as well as spawning oogenetic follicles. The maturation of oogenetic follicles and active gametogenesis of spermatogenic follicles are related to dissolved oxygen and conductivity, and inversely related to temperature (Figure 7). The proportion of oocytes larger than 100 μm is not clearly associated with the analyzed variables. Temperature is strongly associated with the increase in individuals brooding larvae in the gills.

## 4. Discussion

The analysis of the population dynamics of *Corbicula fluminea* and *C. largillierti* in the Santa Catalina stream highlights contrasting adaptive responses to hydrological parameters. The population parameters observed during the study period are consistent with the variable environmental conditions of this system, characterized by recurrent droughts and floods. Over a 10-year period (1997–2007), 499 days of drought emergency were recorded, along with multiple flooding events [37]. Floods generally occur in autumn and winter, when the water table is high, evapotranspiration is low, and human activities, particularly agriculture, leave soils without vegetation cover, thereby increasing surface runoff within the basin [37].

Such environmental instability in streams is known to be a key regulator of freshwater biodiversity, particularly in bivalves. Freshwater organisms commonly exhibit strategies to cope with extreme events; however, the unpredictability of stream hydrology can disrupt dispersal, reproduction, and survival [49].

Regarding the population densities of both *Corbicula* species in the Santa Catalina stream, *C. largillierti* reached higher densities than *C. fluminea* during the first three months of the year. This finding contrasts with observations from other invaded environments where both species coexist, in which *C. fluminea* consistently dominates numerically and attains substantially higher population densities than *C. largillierti* [16,18].

From 2004 onwards, however, density patterns in the Santa Catalina stream became comparable to those reported elsewhere, with both species exhibiting synchronized fluctuations in abundance and *C. fluminea* remaining numerically dominant [18].

The temporary dominance of *C. largillierti* observed in 2003 may be explained by the distinct responses of the two species to environmental variation. Canonical correspondence analysis revealed a common environmental framework characterized by a primary thermal gradient and a secondary hydrological gradient. However, the species differed in the way reproductive traits were associated with hydrological conditions. In *C. largillierti*, gonadal maturation and oocyte growth were more closely related to increases in water level. In contrast, reproductive traits in *C. fluminea* were more strongly associated with stream flow, potentially promoting population growth under the regular flow conditions that prevailed during subsequent non-flood years. These results suggest that, although both species respond to similar environmental drivers, differences in the relative importance of hydrological variables may contribute to the temporal shifts in dominance observed between the populations of *Corbicula* species in the Santa Catalina stream.

*Corbicula fluminea* exhibits a more efficient life-history strategy in this ecosystem, characterized by faster growth and larger body size compared to *C. largillierti*. Together with its high reproductive and adaptive capacity, this supports its invasive success. In contrast, *C. largillierti* displays a slower and more conservative growth pattern. These differences are supported by size-frequency analyses. The dominant size class throughout the sampling period was consistently larger in *C. fluminea*. This agrees with Hünicken et al. [22], who showed that growth investment in *C. fluminea* is directed toward increasing shell size, whereas in *C. largillierti* it is more associated with shell thickening. Additionally, *C. fluminea* exhibited predictable growth over time, with cohorts fitting regression models, whereas *C. largillierti* showed greater variability and stronger cohort overlap. This suggests slower growth after reaching sexual maturity and greater sensitivity to environmental fluctuations such as floods and droughts.

Under typical population dynamics, an increase in individual size within a cohort is associated with a decrease in density [20]. However, in the Santa Catalina stream, hydrological variables obscure this pattern, and neither species shows a clear density trend over time. Population structure was dominated by individuals of similar size, derived from an exceptional recruitment event occurring under short-lived favorable conditions. This cohort constituted the majority of the population throughout the study period. Similar dynamics have been observed in other taxa inhabiting unstable aquatic environments of the region as adaptive mechanism to sustain viable populations. For example, the fish *Prochilodus lineatus* (Valenciennes, 1836) relies on flood-generated nursery areas for reproductive success, rather than larval abundance [50]. Consequently, cohorts originating in flood years dominate population structure until the next major recruitment event.

Given that the distribution of invasive bivalves is closely linked to water levels [35] the dynamics of *Corbicula* spp., particularly *C. largillierti*, despite their phylogenetic and reproductive differences, show population strategies that parallel those described for *P. lineatus*. In this system, fluctuations in water level may act as a demographic bottleneck. Paschoal et al. [51] reported that changes in water column height affect density, distribution, and mean shell length in *C. fluminea*. Under this framework, extreme drought and flood events likely limit the survival of reproductive individuals, particularly those exceeding 20 mm in *C. fluminea* and 12 mm in *C. largillierti*.

Population structure traits are linked to episodic recruitment dynamics, reflected in size-frequency histograms that are unimodal and dominated by cohorts recruited under favorable conditions. In this context, environmental constraints explain population reductions, while the proposed demographic dynamics determine the survival of individuals smaller than 20 mm. The impact of hydrological instability is evident when comparing maximum sizes: while in stable environments *C. fluminea* can reach 37 mm and *C. largillierti* 31 mm [20], in the Santa Catalina stream they do not exceed 28 mm and 20 mm, respectively. These sizes suggest that local populations do not exceed two years of age.

Understanding the reproductive biology of invasive bivalves is essential to explain their establishment, spread, and ecological impact [52]. Populations of *C. fluminea* in Asia, North America, and Africa exhibit two reproductive periods per year (spring and autumn), a lifespan of 1.5–3 years, and rapid juvenile growth [53]. In the Santa Catalina stream, however, no clear pattern of gamete release or reproductive behavior was detected [34]. Mature and developing oocytes were observed throughout the year, even under unfavorable conditions. This allows *C. fluminea* to release gametes opportunistically when conditions become favorable and to rapidly replace them [34]. Indeed, more spawning events were recorded for *C. fluminea* than for *C. largillierti*.

In contrast, identifying reproductive patterns in *C. largillierti* proved difficult, as reported for other *Corbicula* species [36]. Under unfavorable conditions, individuals showed predominantly mature female follicles with a high proportion (>90%) of large oocytes, and few or no developing oocytes. During periods of environmental stability, female gonads contained both mature and developing oocytes, similar to *C. fluminea*, although in lower proportions.

Both species exhibit early parental care through branchial incubation of larvae. *C. largillierti* shows a higher incubation capacity than *C. fluminea* [22]. However, no incubating individuals were observed in either species during flood conditions. Following periods of relative environmental stability, both species showed larval incubation, although it was more frequent in *C. fluminea*.

## 5. Conclusions

Hydrological parameters in the Santa Catalina stream strongly constrained the population performance of *Corbicula fluminea* and *C. largillierti*. These findings indicate that hydrological parameters can act as a limiting factor for the establishment and persistence of invasive *Corbicula* populations. Under fluctuating conditions, *C. fluminea* displayed a more resilient life-history strategy, characterized by greater longevity and larger maximum size, suggesting enhanced survival under environmental stress. In contrast, *C. largillierti* exhibited a strategy biased toward earlier maturation, and reduced maximum size.

Overall, our results demonstrate that the dominance of *C. fluminea* is likely driven by its higher adaptive and reproductive capacity, with opportunistic gamete release throughout the year, allowing it to maintain population stability under hydrological variable regimen, while *C. largillierti* appears to be more sensitive to environmental fluctuations. This differential response highlights the role of environmental parameters as a key factor in species performance and competitive outcomes in invaded freshwater systems. Therefore, this study may have important implications for understanding the mechanisms by which competitive hierarchies among cogeneric invasive species are mediated not only by intrinsic biological traits but also by the environmental context. These results would support monitoring programs, considering that artificial flow management could potentially serve as a control tool for a bioinvasion such as a *Corbicula fluminea* invasion.

## Figures and Tables

**Figure 6:**
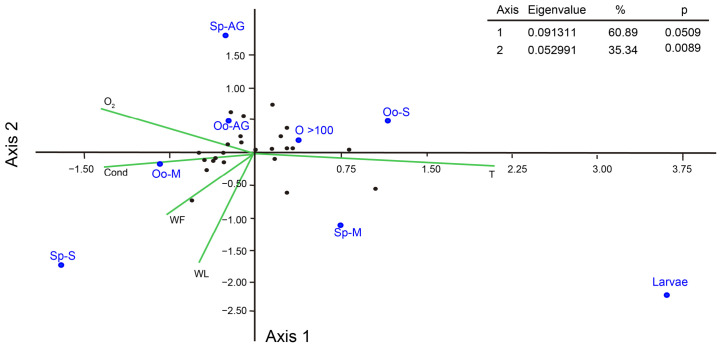
Canonical correspondence analysis triplot (scaling type 2). Black dots, samples. Blue dot, reproductive characteristics of *Corbicula largillierti*: O > 100, oocytes bigger than 100 um; Larvae, proportion of individuals brooding larvae in the gills; Oo-AG, oogenic follicles in active gametogenesis; Oo-M, mature oogenic follicles; Oo-S, spawned oogenic follicles; Sp-AG, spermatogenic follicles with active gametogenesis; Sp-M, mature spermatogenic follicles; Sp-S, spawned spermatogenic follicles. Green lines, environmental variables: Cond, conductivity (uS/cm); WL, mean water level (m); O_2_, oxygen concentration (mg/L); WF, water flow (m^3^/s); T, temperature (°C).

**Table 1:** Environmental variables considered in this study and sampling dates in the Santa Catalina stream [34,36]. Average rainfall (mm); Cond., conductivity (mS); O_2_ (mg/L), O_2_ dissolved (mg/L); O_2_ %, percentage of O_2_; pH, T °C, temperature; TDS, total dissolved solids (mg/L); WF, water flow (m^3^/s); WL, water level (m).

Date	T °C	Cond.	TDS	O_2_ %	O_2_	pH	WL	WF	Average Rainfall
25 January 2003	24.6	560	290	107.8	8.8	7.95			1.87
27 February 2003	26.5	549	278	87.6	6.9	7.67			3.81
31 March 2003	19	578	292	118.5	10.96	8.31			1.97
26 April 2003	13.8	566	285	93.8	9.26	7.74	0.42	0.71	2.78
3 June 2003	8.2	613	307	95.1	11.86	7.69	0.10	0.08	2.13
21 June 2003	11						0.42	0.72	0
19 July 2003	10.2	627	315	125.3	13.39	8.08	0.16	0.15	0.28
12 August 2003	10.5	721	364	98	6.7	7.91	0.20	0.22	3.87
30 September 2003	14	556	277	85	8.8	7.91	0.09	0.06	1.97
27 October 2003	16.9	401	197	57	5.4	7.66	0.08	0.05	3.30
29 November 2003	20.3			66	5.7	7.9		0.28	4.81
18 December 2003								2.26	4.40
15 January 2004							0.22	0.27	2.53
2 February 2004	25	528	268	87	7.4	7.87	0.21	0.23	2.23
23 February 2004	18.6	578	283	36	3.4	7.4	0.18	0.18	4.23
20 March 2004	23.4	544	274	72	6.2	7.65	0.15	0.14	0.76
30 April 2004	11.8		265	70	7.5	7.55	0.22	0.25	4.73
31 May 2004	9.6	543	297	89	10.2	7.43	0.20	0.21	0.32
26 June 2004	15.2	537	280	83	8.4	8.6	0.17	0.17	1.45
31 July 2004	9	530	264	85	9.9	8.17	0.16	0.16	3.52
28 August 2004	13.1	571		93	9.9	7.62	0.19	0.20	3.09
30 September 2004	13.8	554		121	12.9	7.77	0.07	0.04	0.97
29 October 2004	19.7	621	312	91	8.3	7.69	0.05	0.02	1.56
8 December 2004	22.6	515	271	86	7.2	7.52	0.08	0.05	4.40
15 February 2005	18.1	429	215	58	5.1	7.69	0.23	0.27	
20 March 2005		613	310	66	6.2	7.98	0.28	0.36	
30 April 2005							0.16	0.15	

**Table 2:** GenBank accession numbers of cytochrome oxidase I (COI) gene sequences from *Corbicula* individuals collected in this study (in bold) and haplotypes registered in South America available in GenBank.

Species	Form	Haplogroup	Genbank Accession Number
*Corbicula largillertti*	FormCS	FW17	AF519508
*Corbicula largillertti*	FormCS	FW17	PP827595
***Corbicula largillertti***	**FormCS**	**FW17**	**PZ379199**
***Corbicula largillertti***	**FormCS**	**FW17**	**PZ379200**
*Corbicula fluminea*	FormAR	FW5	PP827663
*Corbicula fluminea*	FormAR	FW5	AF519507
***Corbicula fluminea***	**FormAR**	**FW5**	**PZ379201**
***Corbicula fluminea***	**FormAR**	**FW5**	**PZ379202**
***Corbicula fluminea***	**FormAR**	**FW5**	**PZ379203**
*Corbicula* sp.	Form C		AF519512
*Corbicula* sp.		FWBra	MH460421

**Table 3:** Percentage of K2P-corrected genetic distances of *Corbicula* spp. recorded in South America. In bold: the sequences obtained in this study.

		1	2	3	4	5	6	7	8
1	*Corbicula largilliertti* FW17								
**2**	**PZ379199**	**0**							
**3**	**PZ379200**	**0**	**0**						
4	*Corbicula* sp. Form C (AF519512)	1.98	**1.98**	**1.98**					
5	*Corbicula* sp. FWBra (MH460421)	1.48	**1.48**	**1.48**	1.65				
6	*Corbicula fluminea* FW5	2.32	**2.32**	**2.32**	2.83	1.15			
**7**	**PZ379201**	**2.32**	**2.32**	**2.32**	**2.83**	**1.15**	**0**		
**8**	**PZ379202**	**2.32**	**2.32**	**2.32**	**2.83**	**1.15**	**0**	**0**	
**9**	**PZ379203**	**2.32**	**2.32**	**2.32**	**2.83**	**1.15**	**0**	**0**	**0**

**Table 4:** Population characteristics of *C. fluminea* and *C. largillierti* in the Santa Catalina stream.

	*Corbicula fluminea* [34]	*Corbicula largillierti* [36]
Size range with the highest survival rate	27–28 mm	19–20 mm
Size at optimal reproductive age (with larval incubation present)	10–17 mm	10–11 mm
Size of individuals with gonads	<8 mm	<6 mm
Minimum size at maturation	8–9 mm	6 mm
Most frequent size range	16–17 mm	11–12 mm

**Table 5:** Patterns in the reproductive cycle of *C. fluminea* and *C. largillierti* populations in the Santa Catalina stream.

Parameter	*Corbicula fluminea* [34]	*Corbicula largillierti* [36]
Size of studied specimens	6–30 mm (mean: 17.6 mm)	6–18 mm (mean: 11.6 mm)
Proportion of specimens with different follicle types	Hermaphroditic individuals predominated (88.90%) over females (10.90%). Males only recorded in October 2004 and January 2005.	Hermaphroditic individuals predominated (75.49%) over females (21.33%). No males were recorded.
Resting periods	Not observed	Not observed
Gonadal cycle	No clear pattern. Major evacuations in spring and summer; minor evacuations in winter and autumn.	No clear pattern. Major evacuations in spring and autumn, minor evacuations in winter and summer. not observed during flooding conditions.
Oocyte spawning dynamics	Seven spawning events. Three major events (spring: September–October 2003 and September–December 2004; summer: February–March 2004). Four minor spawning events occurred between major events.	Five spawning events. Three major events (autumn: April–May 2004; March 2005; spring: November–December 2004). Additional minor spawning events were recorded.
Reproductive strategy under unstable environmental conditions (droughts and floods)	Great evacuation of mature oocytes was observed, along with minor evacuation events.	Evacuations were less frequent and of lower intensity.
Incubating larvae	Incubating individuals was recorded after intense evacuation events (October 2003: 82.1%; December 2004: 94.3%; February 2004: 60.0%). During droughts and floods periods, larvae were still observed.	Detected after most evacuation events. The highest proportion of incubating individuals was associated with intense evacuation events (December 2004: 45.0%; February 2005: 75.0%; March 2005: 78.6%). No incubating larvae were observed during droughts and floods periods.

## Data Availability

The raw data supporting the conclusions of this article will be made available by the authors upon request.

## Supplementary Materials

The following supporting information can be downloaded at: https://www.mdpi.com/article/10.3390/biology15131026/s1. Figure S1. Size frequency (percentage) measured as maximum anteroposterior length (APL) in mm, of *Corbicula fluminea* in the Santa Catalina stream, Buenos Aires Province, during the sampling period. Specimens smaller than 3 mm are not considered. Figure S2. Size frequency (percentage) measured as maximum anteroposterior length (APL) in mm, of *Corbicula largillierti* in the Santa Catalina stream, Buenos Aires Province, during the sampling period. Specimens smaller than 3 mm are not considered.
